# Animal and Cellular Models of Alzheimer’s Disease

**DOI:** 10.3390/biomedicines12061308

**Published:** 2024-06-13

**Authors:** David Baglietto-Vargas, Kristine K. Freude, Juan Antonio Garcia-Leon

**Affiliations:** 1 Departament Biologia Celular, Genetica y Fisiologia, Instituto de Investigaciones Biomedicas de Malaga-Plataforma BIONAND, Facultad de Ciencias, Universidad de Malaga, 29071 Malaga, Spain; d.baglietto@uma.es; 2 CIBER de Enfermedades Neurodegenerativas, Instituto de Salud Carlos III, Spain; 3 Department of Veterinary and Animal Sciences, Faculty of Health and Medical Sciences, University of Copenhagen, 1870C Frederiksberg, Denmark; kkf@sund.ku.dk

Animal and cellular models have been essential tools over the years to understand many pathogenic mechanisms underlying different neurodegenerative disorders (NDDs), including Alzheimer’s disease (AD) [1,2]. AD is a major NDD impacting the elderly population worldwide. Therefore, generating new models is key to unravelling novel pathogenic processes in order to translate these findings into clinical applications, as well as to advance disease-modifying and preventive therapies [3,4]. This Special Issue focuses on such key pathological processes, including vascular damage, myelinization, sex differences, and metabolic alterations, which could be central in advancing our understanding of the many factettes in AD pathology.

The brain vasculature has a key role in the development and progression of AD. Particularly, alterations at different levels have been described for AD, as these patients present with hypoperfusion (reduced blood flow) in several brain regions and most of them develop cerebral amyloid angiopathy (CAA), a condition characterized by the deposition of amyloid-beta (Aβ) protein in the walls of cerebral blood vessels, associated with an increased risk of cerebral hemorrhage and cognitive decline [5]. Both phenomena are associated with blood–brain barrier (BBB) dysfunction, altering the blood–brain communication and contributing to the increase in brain inflammation and the poor drainage of waste deposited within brain parenchyma [6].

In this issue, Shibly and colleagues investigate early cerebral small vessel changes in the amyloidogenic J20 mouse model of AD [7]. They identified an early decrease in claudin-5 expression (an integral aspect of the tight junctions between endothelial cells that compose the BBB), which was associated with increased expression of vascular endothelial growth factor (VEGF), suggesting an upregulation of angiogenesis, which may impair vascular permeability. In fact, the authors described albumin and Aβ extravasation from the brain parenchyma as early as 3 months of age in these mice. These findings suggest that Aβ may directly affect brain vasculature, further contributing to disease progression by facilitating the infiltration of peripheral immune components into the brain. As these changes start to appear as early as 3 months, it could be suggested that brain blood vessel alterations may be an initial driver of the disease.

Associated with the involvement of the vasculature and BBB dysfunction in AD, astrocytes play a key role in these processes; through their interactions with the brain microvasculature, astrocytes can modulate the entry of circulating factors into the central nervous system (CNS), further supporting their importance as regulators of brain function and blood–brain interactions [8]. In this issue, Amro et al. [9] explored the expression of Aquaporin (AQP) channels (which regulate water homeostasis and osmotic balance) in the human brain and their association with age and AD status, analyzing the transcriptome data from AD and healthy controls from the Allen Brain Atlas database. They found that the expression of AQP channel subtypes in the human brain is more diverse than previously thought and is influenced by both age and AD status. Three classes of AQPs (0, 6, and 8) were found to be upregulated in AD compared to young controls and are associated with reactive oxygen stress and neurodegenerative disease risk. Their findings suggest that targeting these differentially expressed AQPs may be a promising strategy in developing therapeutic interventions that could help mitigate the effects of oxidative stress associated with AD.

The pathology of AD is very complex, with all CNS cell types implicated. Among them, microglia (the professional macrophages of the brain) have a key role in the development and progression of the disease. The key to elucidating their role in the disease—already having occurred a decade ago—was the discovery that mutations in the Trem2 receptor significantly increase the risk of developing sporadic AD [10]. Since then, driven by a significant scientific effort, our comprehension of the importance of microglia and the role of Trem2 in AD has increased dramatically, yet we are still far from understanding their precise contexts in AD. In this Special Issue, Biundo and colleagues [11] investigate the role of Trem2 in a demyelination model (the Csf1r^+/−^ mouse model of Colony-Stimulating Factor-1 Receptor-related Leukoencephalopathy (CRL)). They found that elevation of Trem2 expression and callosal demyelination occur in 4–5-month-old Csf1r^+/−^ mice, prior to the development of symptoms. Therefore, they evaluated how the absence of Trem2 interferes with these phenomena. They found that the absence of Trem2 in Csf1r^+/−^ mice attenuated myelin pathology and normalized microglial densities and morphology in the corpus callosum. The absence of Trem2 also prevented axonal degeneration and the loss of cortical layer V neurons observed in Csf1r^+/−^ mice, as well as preventing the accumulation of myelin-derived lipids in Csf1r^+/−^ macrophages, and reduced the production of TNF-α after myelin engulfment. The study suggests that Trem2 contributes to microglial dyshomeostasis in CRL and indicates the many aspects in which Trem2 and microglia are implicated—key for a thorough understanding of these components in AD—which could translate to the development of effective therapies.

AD is more prevalent in women than men, but we are still far from understanding the causes of this. To address this concern, Kadlecová and colleagues [12] undertook a bibliographical review of research examining the complexity of sex differences, focusing on microglia, and how these differences may impact the risk of developing AD. Although still unknown, the “estrogen hypothesis” establishes that the decline in estrogen levels following menopause, accompanied by the concomitant loss of the neuroprotective effect of estradiol, contributes to the increased vulnerability of women to AD. Furthermore, sex differences in brain aging mechanisms and immune responses, including differences in microglial activity and inflammatory gene expression, also contribute to the greater susceptibility of women to AD. Sex differences in microglia have been reported regarding morphology, functional activity, and gene expression. For instance, microglia from males generally exhibit a more reactive phenotype, with larger soma and increased motility, whereas microglia from females tend to display a more phagocytic phenotype, with gene expression profiles consistent with repair and inflammation regulation. Additionally, females often show higher levels of inflammation-related gene expressions compared to males, particularly in the context of aging. Finally, the authors establish that more relevant models of AD are needed, as the characterization of sex differences in AD models based on human-induced pluripotent stem cell-derived cells and brain organoids. They propose that the use of these models and their exposure to relevant hormones may shed light on the causes of the differential susceptibility of women and men to the disease.

Lipid composition and metabolism is another important field which is emerging in the AD field. Understanding how the lipid balance affects inflammation and brain functioning is central for neurodegenerative conditions. In this issue, Ameen et al. [13] revised the role that short- and medium-chain fatty acids (SCFAs and MCFAs) may play in AD. AD is increasingly being characterized as a metabolic disorder, and metabolic interventions have the potential to reverse symptoms of the disorder. The manuscript reveals important evidence that both SCFAs and MCFAs are effective in reducing Aβ accumulation, while MCFAs and ketone bodies restore the function of mitochondrial complexes and reduce neuroinflammation and glia activation. Therefore, the abundance and composition of SCFAs and MCFAs may have key influences on glial activation, metabolism, and Aβ accumulation. A proper understanding of lipid homeostasis in context-dependent and different cell types is essential to unravel novel pathways implicated in the disease.

As outlined during this Special Issue, an important heterogeneity in terms of cell types, pathogenic processes, and external factors may be implicated in the development and progression of AD. In addition, increasing evidence suggests that peripheral homeostasis and inflammation are closely associated with NDDs. To cover this aspect, López-Villodres and colleagues [14] revised recent and relevant studies on how microbiome alterations (dysbiosis) may provoke immune dysregulation, impacting neuroinflammation through peripheral–central immune communication. They provide key references that demonstrate the impact of dysbiosis on glial cells and neuroinflammation, amyloid deposition, tau pathology, and neurodegeneration in animal models of neurodegenerative conditions, providing insights into the crosstalk between gut microbiota and peripheral and central immune systems, connecting potential links between metabolic diseases, such as obesity and type 2 diabetes, and their association with neurodegenerative diseases, particularly AD. Finally, they suggest that future therapeutic strategies for AD may involve a combination of multitarget drugs in line with the concept of personalized medicine.

Overall, this Special Issue contains a series of intriguing studies that address key questions in the field of AD by using fundamental animal and cellular models for AD, contributing to a better understanding of AD pathology that may guide the future development of effective therapies.

## Data Availability

Not applicable.
